# Crystal structure of 1-nitro-4-(tri­methyl­silylethyn­yl)naphthalene

**DOI:** 10.1107/S2056989015007173

**Published:** 2015-04-15

**Authors:** Jun Du, Graeme J. Moxey

**Affiliations:** a School of Chemical and Material Engineering, Jiangnan University, Wuxi, 214122, People’s Republic of China; b Research School of Chemistry, Australian National University, Canberra, ACT 2601, Australia

**Keywords:** crystal structure, tri­alkyl­silyl­acetyl­ene, nitro­arene, π–π inter­actions

## Abstract

In the title compound, C_15_H_15_NO_2_Si, the dihedral angle between the nitro group and the mean plane of the naphthalene system is 22.04 (11)°. In the crystal, π–π inter­actions generate supra­molecular chains propagating along the *a-*axis direction; the centroid-to-centroid distances range from 3.5590 (12) to 3.8535 (12) Å.

## Related literature   

For the syntheses of aryl­alkynes by Sonogashira coupling, see: Takahashi *et al.* (1980 ▸). For desilylation of the related 1-nitro-4-(tri­methyl­silylethyn­yl)benzene and its use in the construction of metal alkynyl complexes with enhanced non-linear optical properties, see: McDonagh *et al.* (1996*a* ▸,*b* ▸, 2003 ▸); Garcia *et al.* (2002 ▸). For related structures, see: Squadrito *et al.* (1990 ▸); Khan *et al.* (2004 ▸).
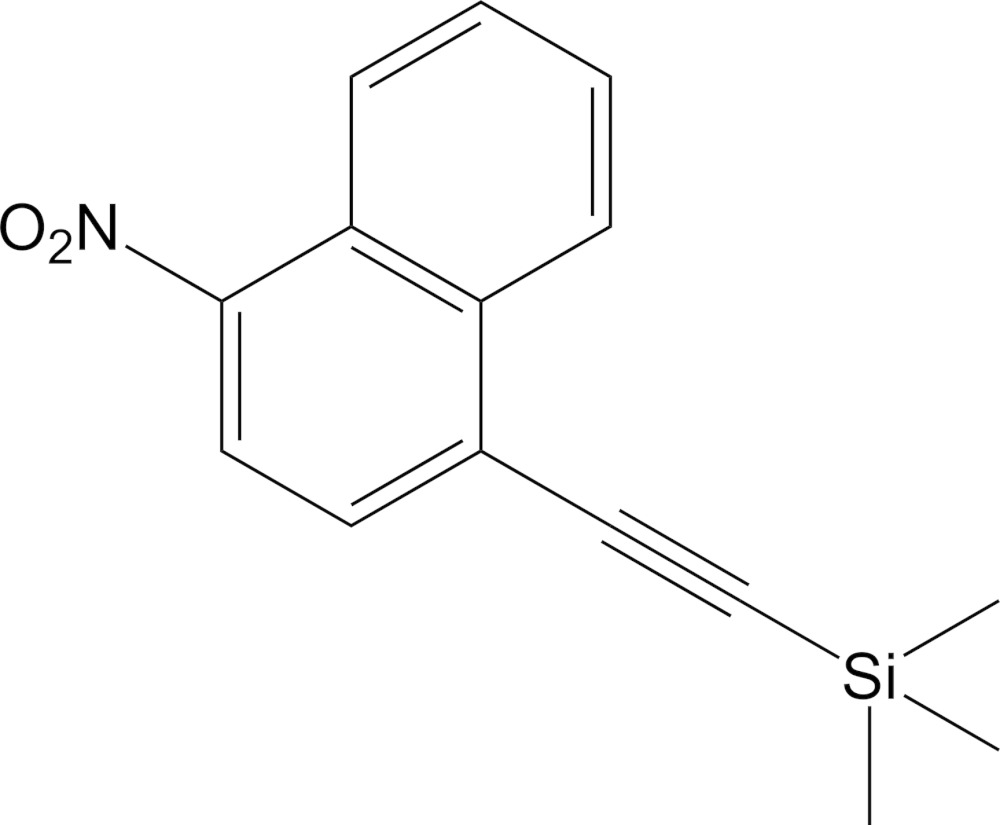


## Experimental   

### Crystal data  

C_15_H_15_NO_2_Si
*M* *_r_* = 269.37
Triclinic, 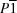

*a* = 6.9679 (9) Å
*b* = 9.2425 (12) Å
*c* = 11.799 (1) Å
α = 100.242 (9)°
β = 99.698 (9)°
γ = 107.127 (12)°
*V* = 694.62 (15) Å^3^
*Z* = 2
Mo *K*α radiation
μ = 0.17 mm^−1^
*T* = 150 K
0.23 × 0.07 × 0.04 mm

### Data collection  

Agilent SuperNova (Dual, Cu at zero, EosS2) diffractometer
Absorption correction: analytical [*CrysAlis PRO* (Agilent, 2014 ▸), based on expressions derived by Clark & Reid (1995 ▸)] *T*_min_ = 0.986, *T*_max_ = 0.996
4695 measured reflections
3112 independent reflections
2621 reflections with *I* > 2σ(*I*)
*R*_int_ = 0.021

### Refinement  

*R*[*F* ^2^ > 2σ(*F* ^2^)] = 0.044
*wR*(*F* ^2^) = 0.114
*S* = 1.07
3112 reflections
175 parameters
H-atom parameters constrained
Δρ_max_ = 0.36 e Å^−3^
Δρ_min_ = −0.23 e Å^−3^

### 

Data collection: *CrysAlis PRO* (Agilent, 2014 ▸); cell refinement: *CrysAlis PRO*; data reduction: *CrysAlis PRO*; program(s) used to solve structure: *SHELXS97* (Sheldrick, 2008 ▸); program(s) used to refine structure: *SHELXL2013* (Sheldrick, 2015 ▸); molecular graphics: *OLEX2* (Dolomanov *et al.*, 2009 ▸); software used to prepare material for publication: *OLEX2*.

## Supplementary Material

Crystal structure: contains datablock(s) global, I. DOI: 10.1107/S2056989015007173/xu5846sup1.cif

Structure factors: contains datablock(s) I. DOI: 10.1107/S2056989015007173/xu5846Isup2.hkl

Supporting information file. DOI: 10.1107/S2056989015007173/xu5846Isup3.cml

**Fig. 1.**
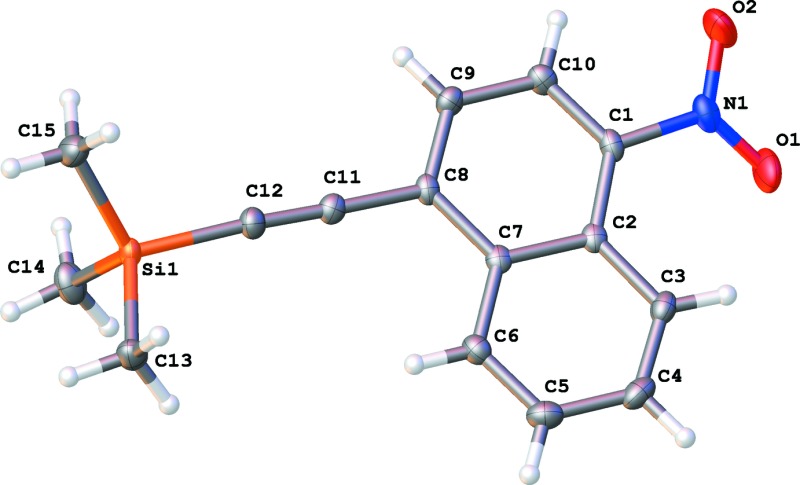
Molecular structure of 1-nitro-4-(trimethylsilylethynyl)naphthalene, with displacement ellipsoids set at the 40% probability level.

. DOI: 10.1107/S2056989015007173/xu5846fig1.tif
Mol­ecular structure of 1-nitro-4-(tri­methyl­silylethyn­yl)naphthalene, with displacement ellipsoids set at the 40% probability level.

**Fig. 2.**
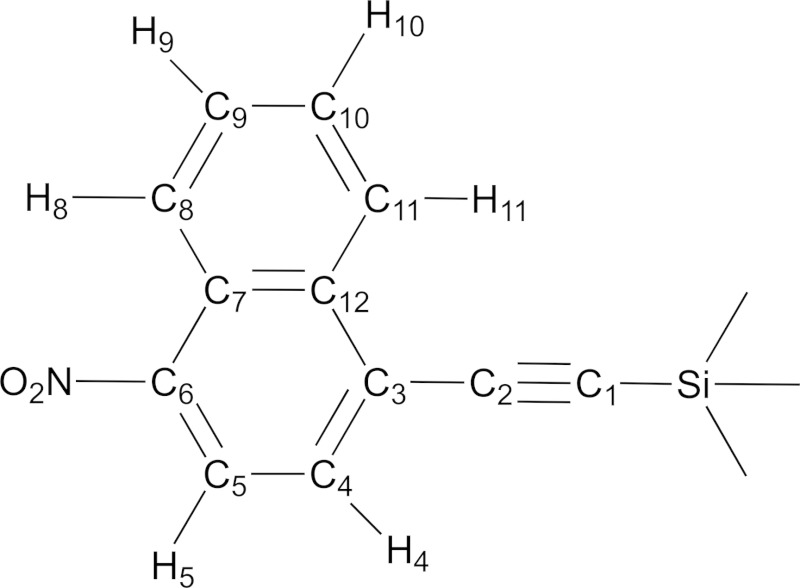
Atom numbering scheme of 1-nitro-4-(trimethylsilylethynyl)naphthalene for 1H and 13C NMR assignments.

1 13 . DOI: 10.1107/S2056989015007173/xu5846fig2.tif
Atom numbering scheme of 1-nitro-4-(tri­methyl­silylethyn­yl)naphthalene for ^1^H and ^13^C NMR assignments.

CCDC reference: 1058939

Additional supporting information:  crystallographic information; 3D view; checkCIF report

## supplementary crystallographic information

### S1. Synthesis and crystallization

1-Iodo-4-nitro­naphthalene (1.196 g, 4.00 mmol) was added to tri­ethyl­amine (30 mL) and the mixture de­oxy­genated and charged with nitro­gen. PdCl_2_(PPh_3_)_2_ (12 mg, 0.016 mmol), CuI (6 mg, 0.03 mmol) and tri­methyl­silyl­acetyl­ene (0.7 mL, 5.00 mmol) were added and the reaction heated to 35 °C overnight. The solution was filtered through filter paper, washing with tri­ethyl­amine (10 mL), and the solvent was removed from the filtrate. The residue was then passed through a short pad of silica, eluting with 4:1 petrol:CH_2_Cl_2_. Reduction in volume of the eluate afforded the product as a yellow solid (1.034 g, 96%). Anal. Calc. for C_15_H_15_NO_2_Si: C, 66.88; H, 5.61; N, 5.20. Found: C, 66.67; H, 5.68; N, 5.28%. ^1^H NMR (δ, 400 MHz, CDCl_3_): 8.55 (d, *J*_HH_ = 8.0 Hz, 1H, H_8_), 8.47 (d, *J*_HH_ = 8.0 Hz, 1H, H_5_), 8.15 (d, *J*_HH_ = 8.0 Hz, 1H, H_11_), 7.79 – 7.65 (m, 3H, H_4_, H_9_, H_10_), 0.36 (s, 9H, Me); ^13^C NMR (δ, 101 MHz, CDCl_3_): 146.3 (C_6_), 134.4 (C_12_), 129.8 (C_9_), 128.9 (C_4_), 128.2 (C_11_), 127.7 (C_3_), 127.1 (C_10_), 125.1 (C_7_), 123.5 (C_8_), 123.3 (C_5_), 105.1 (C_2_), 101.4 (C_1_), 0.1 (s, Me); IR (ATR, cm^-1^): 2956, 2156, 1507, 1323. Bright yellow crystals of the title compound were obtained by diffusion of methanol into a di­chloro­methane solution.

### S2. Refinement

Crystal data, data collection and structure refinement details are summarized below.

### Crystal data

**Table:**

C_15_H_15_NO_2_Si	*Z* = 2
*M**_r_* = 269.37	*F*(000) = 284
Triclinic, *P*1	*D*_x_ = 1.288 Mg m^−^^3^
*a* = 6.9679 (9) Å	Mo *K*α radiation, λ = 0.71073 Å
*b* = 9.2425 (12) Å	Cell parameters from 1967 reflections
*c* = 11.799 (1) Å	θ = 2.6–28.3°
α = 100.242 (9)°	µ = 0.17 mm^−^^1^
β = 99.698 (9)°	*T* = 150 K
γ = 107.127 (12)°	Needle, yellow
*V* = 694.62 (15) Å^3^	0.23 × 0.07 × 0.04 mm

### Data collection

**Table:**

Agilent SuperNova (Dual, Cu at zero, EosS2) diffractometer	3112 independent reflections
Radiation source: SuperNova (Mo) X-ray Source	2621 reflections with *I* > 2σ(*I*)
Mirror monochromator	*R*_int_ = 0.021
Detector resolution: 8.1297 pixels mm^-1^	θ_max_ = 29.2°, θ_min_ = 1.8°
ω scans	*h* = −6→9
Absorption correction: analytical [*CrysAlis PRO* (Agilent, 2014), based on expressions derived by Clark & Reid (1995)]	*k* = −11→12
*T*_min_ = 0.986, *T*_max_ = 0.996	*l* = −15→15
4695 measured reflections	

### Refinement

**Table:**

Refinement on *F*^2^	Primary atom site location: structure-invariant direct methods
Least-squares matrix: full	Hydrogen site location: inferred from neighbouring sites
*R*[*F*^2^ > 2σ(*F*^2^)] = 0.044	H-atom parameters constrained
*wR*(*F*^2^) = 0.114	*w* = 1/[σ^2^(*F*_o_^2^) + (0.0415*P*)^2^ + 0.3469*P*] where *P* = (*F*_o_^2^ + 2*F*_c_^2^)/3
*S* = 1.07	(Δ/σ)_max_ < 0.001
3112 reflections	Δρ_max_ = 0.36 e Å^−^^3^
175 parameters	Δρ_min_ = −0.22 e Å^−^^3^
0 restraints	

### Special details

**Table:**

Experimental. Absorption correction: CrysAlis Pro (Agilent Technologies, 2014) Analytical numeric absorption correction using a multifaceted crystal model based on expressions derived by R.C. Clark & J.S. Reid. (Clark & Reid, 1995). Empirical absorption correction using spherical harmonics, implemented in SCALE3 ABSPACK scaling algorithm.
Geometry. All e.s.d.'s (except the e.s.d. in the dihedral angle between two l.s. planes) are estimated using the full covariance matrix. The cell e.s.d.'s are taken into account individually in the estimation of e.s.d.'s in distances, angles and torsion angles; correlations between e.s.d.'s in cell parameters are only used when they are defined by crystal symmetry. An approximate (isotropic) treatment of cell e.s.d.'s is used for estimating e.s.d.'s involving l.s. planes.

### Fractional atomic coordinates and isotropic or equivalent isotropic displacement parameters (Å^2^)

**Table:**

	*x*	*y*	*z*	*U*_iso_*/*U*_eq_	
C1	0.2485 (3)	0.6318 (2)	0.44838 (15)	0.0204 (4)	
C2	0.1929 (2)	0.4663 (2)	0.41137 (15)	0.0188 (4)	
C3	0.1112 (3)	0.3754 (2)	0.29263 (16)	0.0247 (4)	
H3	0.0875	0.4239	0.2316	0.030*	
C4	0.0675 (3)	0.2178 (2)	0.26757 (17)	0.0293 (4)	
H4	0.0147	0.1606	0.1893	0.035*	
C5	0.1001 (3)	0.1400 (2)	0.35659 (18)	0.0293 (4)	
H5	0.0661	0.0321	0.3376	0.035*	
C6	0.1819 (3)	0.2230 (2)	0.47120 (17)	0.0234 (4)	
H6	0.2048	0.1711	0.5301	0.028*	
C7	0.2324 (2)	0.3874 (2)	0.50191 (15)	0.0182 (4)	
C8	0.3257 (3)	0.4749 (2)	0.62159 (15)	0.0187 (4)	
C9	0.3745 (3)	0.6352 (2)	0.65052 (15)	0.0220 (4)	
H9	0.4329	0.6912	0.7288	0.026*	
C10	0.3369 (3)	0.7131 (2)	0.56329 (16)	0.0225 (4)	
H10	0.3720	0.8211	0.5832	0.027*	
C11	0.3756 (3)	0.3974 (2)	0.71220 (15)	0.0215 (4)	
C12	0.4216 (3)	0.3354 (2)	0.78877 (16)	0.0235 (4)	
C13	0.4757 (3)	0.0381 (2)	0.83030 (17)	0.0300 (4)	
H13A	0.3530	−0.0142	0.7682	0.045*	
H13B	0.4823	−0.0237	0.8873	0.045*	
H13C	0.5949	0.0523	0.7973	0.045*	
C14	0.2518 (3)	0.2089 (3)	0.97894 (19)	0.0361 (5)	
H14A	0.1234	0.1699	0.9203	0.054*	
H14B	0.2647	0.3083	1.0269	0.054*	
H14C	0.2547	0.1369	1.0281	0.054*	
C15	0.7217 (3)	0.3443 (2)	1.00937 (18)	0.0327 (5)	
H15A	0.7507	0.2860	1.0658	0.049*	
H15B	0.7161	0.4421	1.0501	0.049*	
H15C	0.8287	0.3633	0.9667	0.049*	
N1	0.2165 (3)	0.7285 (2)	0.36481 (15)	0.0275 (4)	
O1	0.0903 (2)	0.6698 (2)	0.27072 (14)	0.0437 (4)	
O2	0.3213 (3)	0.86743 (19)	0.39546 (15)	0.0532 (5)	
Si1	0.47002 (8)	0.23164 (6)	0.90416 (4)	0.02062 (14)	

### Atomic displacement parameters (Å^2^)

**Table:**

	*U*^11^	*U*^22^	*U*^33^	*U*^12^	*U*^13^	*U*^23^
C1	0.0176 (8)	0.0279 (10)	0.0240 (9)	0.0126 (7)	0.0093 (7)	0.0138 (7)
C2	0.0125 (7)	0.0262 (9)	0.0212 (8)	0.0085 (7)	0.0063 (7)	0.0086 (7)
C3	0.0193 (9)	0.0349 (11)	0.0191 (9)	0.0081 (8)	0.0036 (7)	0.0076 (8)
C4	0.0228 (9)	0.0372 (12)	0.0213 (9)	0.0066 (8)	0.0030 (8)	−0.0012 (8)
C5	0.0256 (10)	0.0240 (10)	0.0350 (11)	0.0071 (8)	0.0065 (8)	0.0016 (8)
C6	0.0211 (9)	0.0239 (10)	0.0280 (9)	0.0093 (7)	0.0067 (8)	0.0093 (8)
C7	0.0123 (7)	0.0238 (9)	0.0212 (8)	0.0077 (7)	0.0064 (7)	0.0074 (7)
C8	0.0160 (8)	0.0264 (9)	0.0199 (8)	0.0114 (7)	0.0078 (7)	0.0100 (7)
C9	0.0217 (9)	0.0262 (10)	0.0193 (8)	0.0103 (7)	0.0065 (7)	0.0033 (7)
C10	0.0233 (9)	0.0229 (9)	0.0267 (9)	0.0124 (8)	0.0105 (8)	0.0073 (7)
C11	0.0196 (8)	0.0267 (10)	0.0208 (9)	0.0105 (7)	0.0070 (7)	0.0054 (7)
C12	0.0248 (9)	0.0274 (10)	0.0217 (9)	0.0110 (8)	0.0078 (7)	0.0083 (7)
C13	0.0396 (11)	0.0241 (10)	0.0272 (10)	0.0113 (9)	0.0099 (9)	0.0058 (8)
C14	0.0410 (12)	0.0438 (13)	0.0373 (11)	0.0215 (10)	0.0217 (10)	0.0197 (10)
C15	0.0364 (11)	0.0296 (11)	0.0282 (10)	0.0100 (9)	−0.0004 (9)	0.0066 (8)
N1	0.0294 (9)	0.0352 (10)	0.0313 (9)	0.0193 (8)	0.0158 (7)	0.0186 (8)
O1	0.0390 (9)	0.0558 (11)	0.0400 (9)	0.0167 (8)	−0.0007 (7)	0.0290 (8)
O2	0.0889 (14)	0.0287 (9)	0.0447 (10)	0.0188 (9)	0.0129 (9)	0.0198 (8)
Si1	0.0252 (3)	0.0222 (3)	0.0169 (2)	0.0097 (2)	0.00518 (19)	0.00766 (19)

### Geometric parameters (Å, º)

**Table:**

C1—C2	1.427 (3)	C10—H10	0.9300
C1—C10	1.366 (3)	C11—C12	1.201 (2)
C1—N1	1.476 (2)	C12—Si1	1.8403 (19)
C2—C3	1.422 (3)	C13—H13A	0.9600
C2—C7	1.430 (2)	C13—H13B	0.9600
C3—H3	0.9300	C13—H13C	0.9600
C3—C4	1.363 (3)	C13—Si1	1.860 (2)
C4—H4	0.9300	C14—H14A	0.9600
C4—C5	1.399 (3)	C14—H14B	0.9600
C5—H5	0.9300	C14—H14C	0.9600
C5—C6	1.362 (3)	C14—Si1	1.862 (2)
C6—H6	0.9300	C15—H15A	0.9600
C6—C7	1.418 (3)	C15—H15B	0.9600
C7—C8	1.430 (2)	C15—H15C	0.9600
C8—C9	1.382 (3)	C15—Si1	1.853 (2)
C8—C11	1.439 (2)	N1—O1	1.215 (2)
C9—H9	0.9300	N1—O2	1.228 (2)
C9—C10	1.390 (2)		
			
C2—C1—N1	122.38 (16)	C12—C11—C8	178.5 (2)
C10—C1—C2	122.82 (16)	C11—C12—Si1	175.42 (17)
C10—C1—N1	114.80 (16)	H13A—C13—H13B	109.5
C1—C2—C7	116.38 (15)	H13A—C13—H13C	109.5
C3—C2—C1	125.72 (16)	H13B—C13—H13C	109.5
C3—C2—C7	117.84 (17)	Si1—C13—H13A	109.5
C2—C3—H3	119.7	Si1—C13—H13B	109.5
C4—C3—C2	120.51 (17)	Si1—C13—H13C	109.5
C4—C3—H3	119.7	H14A—C14—H14B	109.5
C3—C4—H4	119.2	H14A—C14—H14C	109.5
C3—C4—C5	121.65 (18)	H14B—C14—H14C	109.5
C5—C4—H4	119.2	Si1—C14—H14A	109.5
C4—C5—H5	120.1	Si1—C14—H14B	109.5
C6—C5—C4	119.74 (18)	Si1—C14—H14C	109.5
C6—C5—H5	120.1	H15A—C15—H15B	109.5
C5—C6—H6	119.6	H15A—C15—H15C	109.5
C5—C6—C7	120.89 (17)	H15B—C15—H15C	109.5
C7—C6—H6	119.6	Si1—C15—H15A	109.5
C2—C7—C8	119.87 (16)	Si1—C15—H15B	109.5
C6—C7—C2	119.33 (16)	Si1—C15—H15C	109.5
C6—C7—C8	120.79 (16)	O1—N1—C1	119.96 (17)
C7—C8—C11	120.25 (16)	O1—N1—O2	123.02 (17)
C9—C8—C7	120.30 (16)	O2—N1—C1	117.02 (17)
C9—C8—C11	119.42 (16)	C12—Si1—C13	107.97 (9)
C8—C9—H9	119.8	C12—Si1—C14	106.63 (9)
C8—C9—C10	120.33 (16)	C12—Si1—C15	109.92 (9)
C10—C9—H9	119.8	C13—Si1—C14	110.88 (10)
C1—C10—C9	120.29 (17)	C15—Si1—C13	109.63 (10)
C1—C10—H10	119.9	C15—Si1—C14	111.70 (10)
C9—C10—H10	119.9		
			
C1—C2—C3—C4	178.69 (16)	C5—C6—C7—C8	−177.53 (16)
C1—C2—C7—C6	−179.69 (14)	C6—C7—C8—C9	179.94 (15)
C1—C2—C7—C8	−0.9 (2)	C6—C7—C8—C11	1.9 (2)
C2—C1—C10—C9	−0.7 (3)	C7—C2—C3—C4	1.7 (2)
C2—C1—N1—O1	21.6 (2)	C7—C8—C9—C10	−1.2 (2)
C2—C1—N1—O2	−158.72 (17)	C8—C9—C10—C1	0.9 (3)
C2—C3—C4—C5	0.2 (3)	C10—C1—C2—C3	−176.32 (16)
C2—C7—C8—C9	1.2 (2)	C10—C1—C2—C7	0.7 (2)
C2—C7—C8—C11	−176.86 (14)	C10—C1—N1—O1	−158.99 (17)
C3—C2—C7—C6	−2.4 (2)	C10—C1—N1—O2	20.7 (2)
C3—C2—C7—C8	176.36 (15)	C11—C8—C9—C10	176.88 (15)
C3—C4—C5—C6	−1.5 (3)	N1—C1—C2—C3	3.1 (3)
C4—C5—C6—C7	0.7 (3)	N1—C1—C2—C7	−179.89 (14)
C5—C6—C7—C2	1.2 (2)	N1—C1—C10—C9	179.83 (14)
