# Supplementary figures and images for: Crystal structure of 1-nitro-4-(tri­methyl­silylethyn­yl)naphthalene
Source: Acta Crystallogr E Crystallogr Commun. 2015 Apr 15;71(Pt 5):o311–2. doi: 10.1107/S2056989015007173 (PMC4420069; doi:10.1107/S2056989015007173)

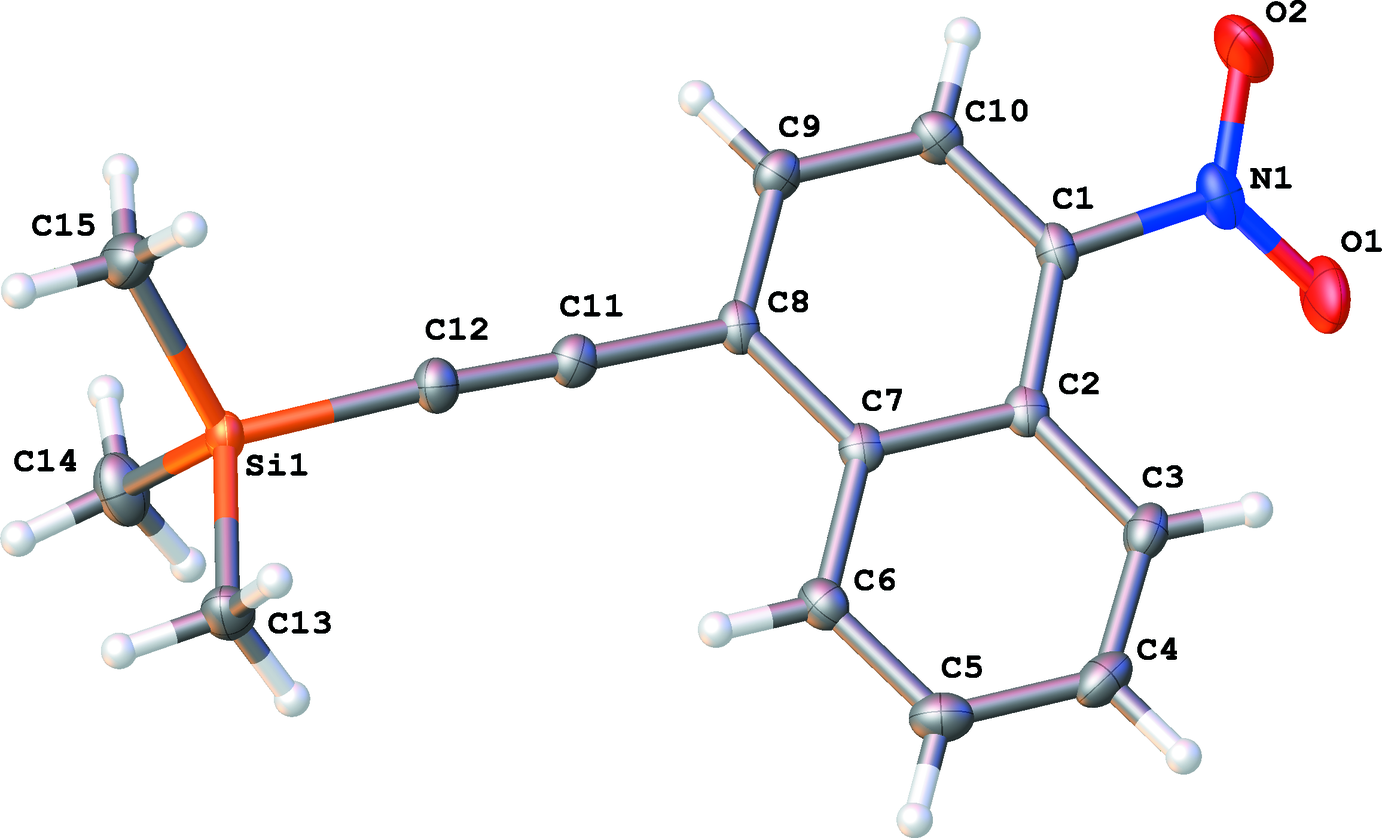

Supplement: Supplementary file 4 [file e-71-0o311-fig1.tif]

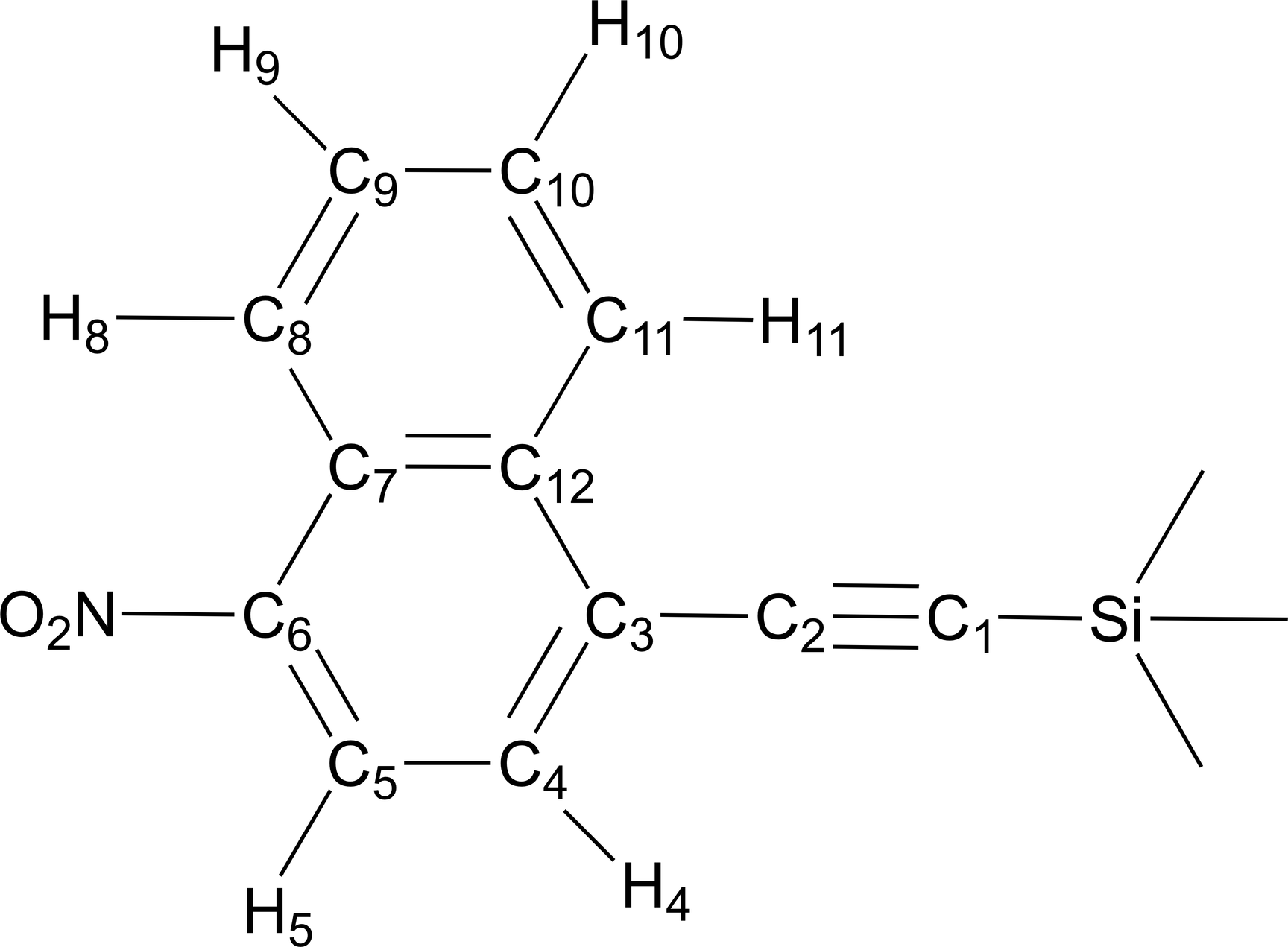

Supplement: Supplementary file 5 [file e-71-0o311-fig2.tif]
